# Use of *mariner* transposases for one-step delivery and integration of DNA in prokaryotes and eukaryotes by transfection

**DOI:** 10.1093/nar/gkx113

**Published:** 2017-02-16

**Authors:** Maryia Trubitsyna, Gracjan Michlewski, David J. Finnegan, Alistair Elfick, Susan J. Rosser, Julia M. Richardson, Christopher E. French

**Affiliations:** 1 Institute of Quantitative Biology, Biochemistry and Biotechnology, School of Biological Sciences, University of Edinburgh, Edinburgh EH9 3FF, UK; 2 Institute of Cell Biology, School of Biological Sciences, Wellcome Trust Centre for Cell Biology, University of Edinburgh, Edinburgh EH9 3BF, UK; 3 Institute of Cell Biology, School of Biological Sciences, University of Edinburgh, Edinburgh EH9 3FF, UK; 4 Institute of BioEngineering, School of Engineering, University of Edinburgh, Edinburgh EH9 3JL, UK; 5 Institute of Quantitative Biology, Biochemistry and Biotechnology, School of Biological Sciences, UK Centre for Mammalian Synthetic Biology, University of Edinburgh, Edinburgh EH9 3FF, UK

## Abstract

Delivery of DNA to cells and its subsequent integration into the host genome is a fundamental task in molecular biology, biotechnology and gene therapy. Here we describe an IP-free one-step method that enables stable genome integration into either prokaryotic or eukaryotic cells. A synthetic *mariner* transposon is generated by flanking a DNA sequence with short inverted repeats. When purified recombinant Mos1 or Mboumar-9 transposase is co-transfected with transposon-containing plasmid DNA, it penetrates prokaryotic or eukaryotic cells and integrates the target DNA into the genome. *In vivo* integrations by purified transposase can be achieved by electroporation, chemical transfection or Lipofection of the transposase:DNA mixture, in contrast to other published transposon-based protocols which require electroporation or microinjection. As in other transposome systems, no helper plasmids are required since transposases are not expressed inside the host cells, thus leading to generation of stable cell lines. Since it does not require electroporation or microinjection, this tool has the potential to be applied for automated high-throughput creation of libraries of random integrants for purposes including gene knock-out libraries, screening for optimal integration positions or safe genome locations in different organisms, selection of the highest production of valuable compounds for biotechnology, and sequencing.

## INTRODUCTION

Targeted genome modifications can be efficiently achieved using the bacterial immunity-derived system CRISPR-Cas9 (1–4). However, in some circumstances alternative methods such as DNA transposition can provide a more appropriate editing tool. For example, if the optimal position for a desired genome integration is not known in advance and has to be determined; or if random integrations are preferred (5), for example to create libraries of clones (6,7); or to sequence new organisms by introducing a known marker in uncharacterized genomes.

DNA transposase enzymes can recognize short, inverted repeat (IR) DNA sequences, excise DNA flanked with IRs (transposon) and integrate it into a new location (8). Transposons have been successfully used to modify genomes of prokaryotic and eukaryotic cells (9,10), and are being used in preliminary clinical trials (11).

Currently DNA transposition—excision followed by integration of a gene—can be achieved by injection or co-transfection of a donor plasmid, bearing a gene of interest flanked by IRs, together with a helper plasmid expressing transposase (12–15). Active transposase, expressed inside the cell recognizes the transposon IRs on the donor plasmid, excises the gene of interest and inserts it into genomic DNA. However, constant expression of the transposase can result in excision of the newly integrated transposons (16), or lead to overproduction inhibition, a feature of *mariner* family transposition (17–20). The *in vivo* activity of the *mariner* transposase Mos1 expressed from a helper plasmid is much lower than that of piggyBac and Sleeping Beauty transposases, the most promising representatives for use in mammalian transgenesis (21). This may be because Mos1 transposase is expressed in high quantities *in vivo* and forms aggregates in cells, reducing the transposition efficiency (20,22).

To solve the problem of constant expression of transposase inside the cell, a single plasmid, carrying both the transposon and the transposase expression genes, can be transfected (23,24). Similar to the helper plasmid method, the potential for recombination of the transposase gene with the host genomic DNA makes this approach unsuitable for gene therapy. Moreover, the transposition rate is dependent upon the intracellular protein expression level, which limits the approach to strains and cell lines that enable sufficient expression levels.

In alternative approaches, purified protein or mRNA of the transposase can be injected into a living cell to promote integration or excision of transposons *in vivo* (25–27). The injection procedure is an invasive technique, which results in low throughput. Transfection of mRNA in mammalian cells was shown to be less efficient than the use of the helper plasmid, possibly due to the short window of expression before the mRNA is degraded (28–30). piggyBac transposase was successfully packaged and delivered to mammalian cells by lentiviral particles, but this delivery approach was not feasible with other transposases (31).

Several commercially available kits use Tn5, Mu or Tn7 transposons for insertional mutagenesis (Epicentre^®^, ThermoFisher Scientific Inc., New England Biolabs^®^ Inc.). Tn5 and Mu were shown to be active for *in vivo* integration, for example, in the widely used EZ-Tn5^™^system, but published protocols require electroporation (32) or microinjection (33,34) and are thus unsuitable for automated high-throughput approaches.

In order to overcome these limitations we have developed a method in which purified recombinant *mariner* transposases, Mos1 and Mboumar-9, deliver and integrate DNA of interest *in vivo* into genomes of bacterial and mammalian cells following facile transfection: chemical transfection, electroporation or Lipofection. This will broaden the spectrum of organisms suitable for *in vivo* mutagenesis, *de novo* sequencing, genome-wide functional screening, since it allows a simple and automatable transfection method to be used to deliver and integrate DNA of interest into the genome.

## MATERIALS AND METHODS

### *In vivo* transposition assay and inhibition

Plasmid DNA (7 nM) carrying the gene of interest and flanked with short IRs (5΄-tatcaggtgtacaagtatgaaatgtcgttt-3΄ for Mos1 transposition or 5΄-taccaggtgtgtcggtaattcctttccggttttt-3΄ for Mboumar-9 transposition) was incubated with 70 nM purified transposase (protein:DNA molar ratio 10:1) for 1 h at 30°C in buffer containing 25 mM 4-(2-hydroxyethyl)-1-piperazineethanesulfonic acid (HEPES) pH 7.5, 100 mM NaCl, 10% v/v glycerol, 2 mM dithiothreitol (DTT), 200 μg/ml acetylated bovine serum albumin, 10 mM MnCl_2_ in final volume 20 μl. The inhibition of the pre-incubation step was performed by addition of proteinase K to a final concentration of 1 mg/ml prior to additional 30 min incubation at 37°C (for Figure 2C). *In vivo* transposition also occurs if the pre-incubation step is omitted, but with ∼10× less efficiency.

For bacterial and mammalian cells chemical transfections the whole reaction volume (20 μl) was used, whereas for electroporation 2 μl of the reaction was used.

### Preparation of chemically competent cells

A single colony was used to inoculate 5 ml Lysogeny Broth (LB) from a freshly streaked plate, and incubated overnight at 37°C with 250 rpm agitation. The culture was diluted 1:200 into 100 ml pre-warmed LB with 20 mM MgCl_2_ and incubated at 37°C with 250 rpm agitation until OD_600_ reached 0.48 for *Escherichia coli* DH10B. The culture was transferred to a chilled 250 ml centrifuge bottle and incubated on ice for 10 min. The cells were harvested for 5 min at 4000 *g* at 4°C. The pellet was resuspended in 40 ml of cold TFB1 buffer (30 mM CH_3_COOK, 100 mM RbCl, 10 mM CaCl_2_, 50 mM MnCl_2_, 15% v/v glycerol, pH 5.8) by gently pipetting and incubated on ice for 5 min. Cells were then pelleted for 10 min at 1400 *g* at 4°C. The pellet was gently resuspended in 4 ml of cold TFB2 buffer (10 mM MOPS, 75 mM CaCl_2_, 10 mM RbCl, 15% v/v glycerol, pH 6.5) per 100 ml of culture and incubated on ice for 15 min. Aliquots of 100 μl were dispensed into pre-chilled 1.5 ml microcentrifuge tubes. Tubes were stored at −80°C after snap freezing in liquid nitrogen.

### Bacterial transfection

Chemically competent *E. coli* DH10B were prepared using the RbCl method, as described above. The transposition reaction (20 μl) was added to the cells thawed on ice, tubes were gently flicked and incubated on ice for 30 min. After 90 s heat shock in a 42°C water bath, the tubes were returned to ice for 2 min and 400 μl of SOC medium (room temperature) was added. The tubes were incubated for 80 min at 37°C and 200 rpm shaking prior to plating on LB agar containing kanamycin 50 μg/ml. The colonies were scored after 20 h incubation at 37°C.

Commercially available competent cells: one Shot^®^ TOP10 Chemically Competent *E. coli* (Invitrogen) (for Figure 3C) and One Shot^®^ TOP10 Electrocomp^™^*E. coli* (for Figure 3A)—were used according to the manufacturers' instructions.

### Protein cloning and purification

Mos1 and Mboumar-9 transposases were purified as previously described (35,36).

The N-terminal Green Fluorescent Protein (GFP) fusion of Mos1 was assembled with pET30a expression vector (Novagen) using the PaperClip DNA assembly method (37). A flexible linker (GGGGS)_3_ was inserted between the GFP and Mos1 sequences according to the published protocol for intervening sequences (37).

Sequence verified plasmids were transformed into *E. coli* BL12 Gold (DE3). Protein expression was induced in Terrific Broth with IPTG at a final concentration of 1 mM at OD_600_ = 0.6 for 21 h at 220 rpm agitation and 25°C. Cells were collected at 8000 *g* for 1 h at +4°C. Five grams of cell pellet was resuspended in 50 ml buffer containing 20 mM NaPO_4_ pH 7.4, 500 mM NaCl, 5 mM MgCl_2_, 1 Kunitz DNase, 400 μg/ml lysozyme, 2 tablets Complete Protease inhibitor cocktail (Roche) and incubated for 1 h at +4°C with agitation. The cell suspension was homogenized by passing through a needle and cells were broken down in a Cell Disruptor (1.1 kW TS) at 25 kpsi. Cell debris was pelleted at 50 000 *g* for 1 h at 4°C. Supernatant was filtered through a 5 μm filter followed by a 0.45 μm filter (Millipore) and imidazole was added to a final concentration of 2 mM, prior to loading onto a HiTrap IMAC FF 1 ml column charged with Ni^2+^. The column was pre-equilibrated with 20 mM NaPO4 pH 7.0, 300 mM NaCl, 10 mM Imidazole. The injection flow rate was 1 ml/min. The column was washed with two column volumes (CV) of equilibration buffer and bound proteins were eluted with a linear gradient from 20 mM NaPO_4_ pH 7.0, 300 mM NaCl, 10 mM Imidazole to 20 mM NaPO_4_ pH 7.0, 300 mM NaCl, 500 mM Imidazole over 10 CV. Fractions (1 ml) were collected and analyzed by sodium dodecyl sulphate-polyacrylamide gel electrophoresis (SDS-PAGE) using 12% polyacrylamide gels. Fractions containing bands of the expected size were pooled and concentrated to 250 μl using a Vivaspin^®^ 6 mwco 10 000 Da. Further purification and analysis was performed on a Superdex 200 10/300 GL column at 0.5 ml/min flow rate. The final protein purity was estimated by ImageLab Software to be 75–80%.

### Southern blotting

Genomic DNA (1 μg) was separated on a 1% (w/v) agarose gel after overnight endonuclease digestion with EcoRI New England Biolabs (NEB). The gel was washed twice with dH_2_O, incubated with the depurination solution (0.2 M HCl) for 20 min with gentle shaking and rinsed with dH_2_O. The DNA was denaturated by 2 × 15 min incubation with 0.5 M NaOH, 1.5 M NaCl with gentle shaking and rinsed with dH_2_O. The solution was neutralized by 30 min incubation in 0.5 M Tris pH 7.5, 1.5 M NaCl with gentle shaking, rinsed with dH_2_O and finally equilibrated for 30 min in 20× Saline Sodium Citrate (SSC) solution (3 M NaCl, 30 mM sodium citrate) with gentle shaking.

Capillary transfer was performed overnight at room temperature: the membrane was then rinsed with dH_2_O and cross-linked for 90 s under UV (UV Stratalinker 1800).

Pre-hybridization was performed in a plastic bag at 55°C for 1.5 h in 8 ml of hybridization solution with 120 rpm agitation. Hybridization was performed at 55°C for 15 h in a plastic bag containing 5 ml of hybridization solution with 3 μl of 100 μM fluorescently labeled 28 bp Mos1 IR (5΄ IRDye^®^ 700, IDT) with 120 rpm agitation. The membrane was washed for 5 min at room temperature in 2× Saline-Sodium Phosphate-Ethylenediaminetetraacetic acid (SSPE), 5 min at 40°C min 2× SSPE, 2× for 15 min at 50°C in 2× SSPE/1% SDS, 2× for 15 min at 50°C in 0.2× SSPE. Fluorescent dye was visualized on a LI-COR Imaging System using Odyssey software.

### Sequencing of prokaryotic genome integration sites

Integrations in the bacterial genome were mapped by two methods: cloning of genomic DNA and inverse polymerase chain reaction (PCR).

For cloning of genomic DNA 5 μg of genomic DNA and 1 μg of pBSKS(+) was digested with EcoRI (NEB) in a final volume 50 μl overnight at 37°C. The enzyme was heat inactivated and 100 ng of pBSKS(+) was ligated with 400 ng of genomic DNA with T4 DNA ligase (NEB) in final volume 10 μl at 4°C overnight. The whole reaction was transformed into *E. coli* DH10B chemically competent cells. After recovery the total volume was plated out on LB agar containing kanamycin (50 μg/ml) and carbenicillin (100 μg/ml). Plasmid DNA was isolated from the resistant clones and analyzed by restriction digest and Sanger sequencing.

Inverse PCR: ∼1 μg of genomic DNA was digested with 1 μl of EcoRI (NEB) in 20 μl for 3 h at 37°C. The endonuclease was heat inactivated for 20 min at 65°C. Two dilutions (1:10 and 1:20) were prepared for self-ligation of the genomic DNA fragments in a final volume of 20 μl at room temperature with Rapid DNA Ligation Kit (Roche) for 20 min.

PCR was performed with Phusion High-Fidelity Polymerase (ThermoScientific) in a final volume of 20 μl, using 400 μM dNTPs (Invitrogen), 500 nM of primers (5΄-gtttcccgttgaatatggctc-3΄ and 5΄-actttctggctggatgatgg-3΄), 1 μl of DNA ligation mixture, 3% v/v Dimethyl sulfoxide (DMSO), 0.2 μl of Phusion Polymerase, dH_2_O to 20 μl. The initial denaturation step was 98°C for 30 s, followed by 30 cycles of 98°C for 30 s, 56°C for 20 s and final elongation of 72°C for 7 min. The whole PCR reaction was analyzed on a 1% (w/v) agarose gel. The brightest bands were cut out, gel purified (Qiagen) and sequenced.

### Construction of transposons

The transposon containing the kanamycin resistance cassette only was constructed previously (38). We used donor plasmids with either two right Mos1 IRs (pEPMosRR) or with two left Mboumar-9 IRs (pEPMboLL) as they were shown to be the most efficient for *in vitro* transposition (38). The SalI site within the backbone pEP185.2 was deleted by site directed mutagenesis using primers 5΄-ccctcgaggtagacggtatcgataagc-3΄ and 5΄-gcttatcgataccgtctacctcgaggg-3΄. The kanamycin cassette was cut out of the plasmid using the remaining two SalI sites, leaving the IRs within the backbone vector. The new inserts were amplified using primers 5΄-atttatgtcgaccgctgaggtctgcctcg-3΄ and 5΄-ttaaatgtcgacggatccaggctcatccagcc-3΄ or 5΄-ttaaatgtcgactctagattcggagtgagc-3΄ and 5΄-taagatgtcgacttcaaatatgtatccgctcatg-3΄ from the plasmid products of three-, four-, five- and six-parts assemblies by PaperClip (37). The larger transposons were obtained in this study by assembly of seven, eight and nine parts in pSB1C3 according to the PaperClip protocol including boost PCR (Supplementary Table S3). These transposons were also amplified with the primers 5΄-ttaaatgtcgactctagattcggagtgagc-3΄ and 5΄-taagatgtcgacttcaaatatgtatccgctcatg-3΄, which introduced flanking SalI sites and were cloned into pre-digested and gel-purified backbone pEP185.2 carrying the IR sequences.

To create the donor plasmid for mammalian cell transfection, the neomycin gene (neoR) under the regulation of the SV40 promoter was used. We deleted the SalI site from pBSKS(+) with site directed mutagenesis using primers 5΄-gcttatcgataccgacgacctcgaggggg-3΄ and 5΄-ccccctcgaggtcgtcggtatcgataagc-3΄ creating pBSKS(+)ΔSalI. Then we subcloned the kanR gene with the IRs sequences from pEPMosRR (digested with XbaI) and from pEPMboLL (digested with SacI) into pBSKS(+)ΔSalI using the appropriate sites. The kanamycin resistance cassette was cut out of both transposons with SalI. The neomycin resistance cassette of 1.6 kb was amplified from the pEGFP-C1 vector with the primers 5΄-tccatagtcgacagtcctgaggcggaaagaacc-3΄ and 5΄-tccatagtcgacatgagtaacctgaggctatggc-3΄ introducing SalI recognition sites. The resulting products were cloned using the SalI sites into the prepared vectors with two right IRs of Mos1 or two left IRs of Mboumar-9 transposons, creating pMosNeo and pMboNeo plasmids. The correct order and absence of mutations of the resulting constructs were confirmed by sequencing.

### Tissue culture

HeLa cell line (ATCC^®^ CCL-2) was maintained in DMEM (Dulbecco's Modified Eagle Medium) (Gibco^®^, No. 41966), containing 10% fetal bovine serum (SIGMA, F7524) and 1% penicillin-streptomycin (SIGMA, P4333).

HEK293-H cell line (Gibco^®^, No. 11631-017) was maintained in DMEM (Gibco^®^, No. 41966), containing 10% fetal bovine serum (SIGMA, F7524) and supplemented with 0.1 mM Minimum Essential Medium (MEM) non-essential amino acids (Gibco^®^, No. 11140).

Cells were inoculated into 6-well plates, one day before transfection in several dilutions (0.8–3.2 × 10^5^ cells per well). For transfection, cells of 60–70% confluence were used. The growth medium was aspirated, and cells were washed with 2 ml of Dulbecco's Phosphate-Buffered Saline (DPBS) (Gibco^®^, No. 14190094), then with 2 ml of Opti-MEM^®^ Reduced Serum (Gibco^®^, No. 31985).

For each transfection reaction, 1 tube containing 245 μl of Opti-MEM^®^ with 3–5 μl of either Lipofectamine^®^ 2000 or GeneJuice^®^ and 1 tube with 230 μl of Opti-MEM^®^ plus the whole volume (20 μl) of the transposition reaction were prepared, and incubated at room temperature for 5 min. (If the volume of the transposition reaction was <20 μl, the volume of Opti-MEM^®^ was increased to maintain the final volume of 250 μl). The solution containing DNA was added drop-wise to the tubes containing transfection reagent (with no vortexing or mixing). The tubes were incubated at room temperature for 30 min and each 500 μl was added drop-wise to 1.5 ml of DMEM in each well. The plate was rocked gently and incubated for 24 h prior to medium changing and/or splitting the cells for selection. For selection G418 was added to a final concentration of 1200 μg/ml for HeLa cells or 1000 μg/ml for HEK293-H cells after 72 h after splitting the cells.

Selection was performed for 10–14 days (until visible colonies were formed). Colonies were fixed in ice-cold methanol for 5–10 min, stained with 0.1% (w/v) brilliant blue in methanol for 15 min and washed with DPBS until a clear background was achieved. For cell counting, cells were dissociated for 5 min with 0.2 ml TrypLE Express (Gibco^®^, No. 12605-010) and the reaction was quenched with 0.5 ml DPBS. Cells were counted with a Countess^™^ Automated cell counter (Invitrogen) according to the manufacturer's protocol.

### Sequencing of eukaryotic genome integration sites

HeLa cells from one well of a 6-well plate were trypsinized and pelleted at 400 *g* for 5 min at room temperature. Genomic DNA was isolated with a Wizard^®^ Genomic DNA purification Kit (Promega). After overnight digestion of 2.5 μg of genomic DNA with HindIII-HF, the restriction enzyme was heat inactivated for 20 min at 80°C. DNA was phenol extracted, ethanol precipitated and dissolved in 20 μl of water, pre-warmed to 70°C. Self-ligation was performed overnight in 100 μl total volume using 150 ng of digested genomic DNA and T4 DNA Ligase (NEB) according to the manufacturer's protocol. Ligated DNA was phenol extracted, ethanol precipitated and dissolved in 20 μl of water pre-warmed to 70°C.

To amplify both junctions flanking the neomycin resistance cassette, inverse PCR was performed using PCR Master Mix (Promega) with the addition of Pfu DNA Polymerase (Agilent Technologies) in a final volume of 50 μl: 25 μl of Master Mix, 160 μM dNTPs, 500 nM of primers (5΄-gaggctaactgaaacacggaaggag-3΄ and 5΄-cgggactatggttgctgactaattg-3΄), 10 μl of ligated DNA and dH_2_O to 50 μl. The initial denaturation step was 95°C for 2 min, followed by 40 cycles of 95°C for 30 s, 63°C for 30 s, 73°C for 3 min and final elongation of 73°C for 5 min. Fifteen microliters of the PCR reaction was analyzed on a 1% (w/v) agarose gel. The brightest bands were cut out, gel purified (Qiagen) and cloned into pJET plasmid using the CloneJET PCR Cloning Kit (ThermoFisher Scientific) according to the manufacturer's protocol. Carbenicillin resistant colonies were selected overnight. Plasmid DNA was isolated and sequenced to establish the locations of neomycin cassette integrations in mammalian cells.

### Live cell imaging

Prior to transfection, cells were inoculated into a MatTek glass bottomed dish (P35GC-1.5-14-C). Following transfection as described above, imaging was performed at 37°C and 5% CO_2_ on a Deltavision Elite microscope with Photometrics Coolsnap CCD camera and objective ×60 PlanApoN NA 1.42 Oil. Images of the cells were taken every 20 min during 61 time points and throughout 10 positions on a dish. Data were analyzed using ImageJ v10.2. Twelve Z-stacks (1 μm), summed and the pixel intensity of a 5 μm circle of the cytoplasm and a 5 μm circle of the nucleus were compared (excluding the points of saturation for the camera). The averages of 10 positions on a dish are presented and standard deviations are shown as shadowed error bars.

### Western blotting

Total protein samples were isolated from cells by sonication in buffer D (20 mM Tris pH 7.9, 20% w/v glycerol, 0.1 M KCl, 0.2 mM Ethylenediaminetetraaceticacid, 0.5 mM DTT, 0.2 mM phenylmethylsulphonyl using Bioruptor Pico (Diagenode) and centrifugation at 5000 *g* for 10 min in 4°C. Subsequently, 250 μg of total protein extracts or recombinant purified proteins (GFP: 1 ng per lane, GFP-Mos1: 10 ng per lane) were separated on 4–12% NuPAGE SDS-PAGE gels using MOPS running buffer (ThermoFisher) and were transferred onto a nitrocellulose membrane (GE Healthcare). The membrane was blocked overnight at 4°C with 1:10 Western Blocking Reagent (Roche) in Tris-buffered saline, Tween 20 (TBST) buffer (20 mM Tris pH 7.5, 137 mM NaCl and 0.1% (v/v) Tween 20). The next day, the membrane was incubated for 1 h at RT with primary rabbit polyclonal anti-GFP (1:1000, 50430-2-AP, Proteintech) solution in 1:20 Western Blocking Reagent diluted in TBST. After washing in TBST, the blots were incubated with the secondary anti-rabbit antibody (1:1000, #7074, Cell Signalling Technology) conjugated to horseradish peroxidase and were detected with WesternSure PREMIUM Chemiluminescent Substrate detection reagent using C-DiGit Blot Scanner (LI-COR).

## RESULTS

### *In vivo* integration in bacterial genomes

**Figure 1.**
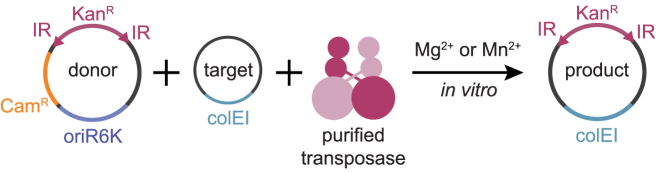
*In vitro* transposition assay (*in vitro* hop assay). A donor plasmid contains a selection cassette (kanamycin resistance, Kan^R^) flanked with the inverted repeats (IR), which are recognized by transposase. Upon addition of divalent ions Mg^2+^ or Mn^2+^*in vitro*, purified transposase excises the selection cassette and integrates it into the target plasmid. Purified products of *in vitro* transposition can be analyzed by transfection into *Escherichia coli* DH10B cells and selection in the presence of kanamycin. The donor plasmid contains a conditional origin of replication oriR6K and will not propagate in the destination strain; only the kanamycin cassette containing target plasmids are maintained.

**Figure 2.**
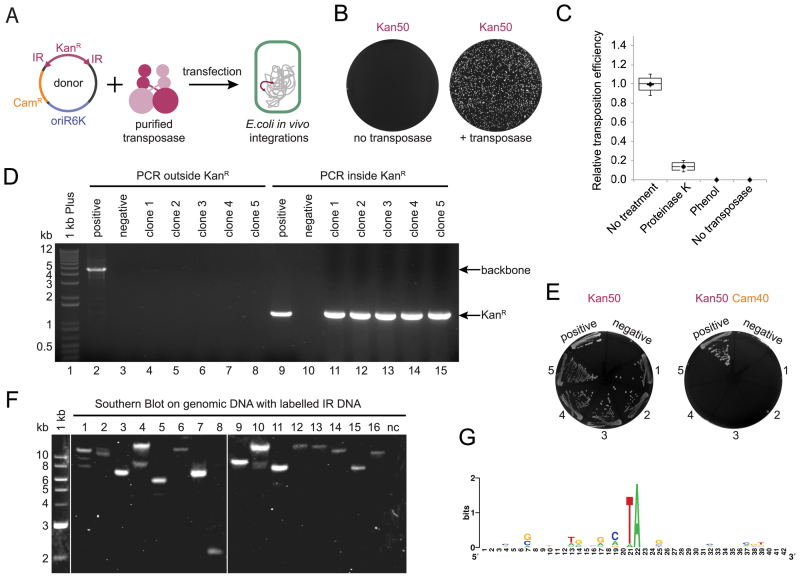
*In vivo* transposition in prokaryotic cells. (**A**) Scheme of the experimental method. The donor plasmid DNA, carrying a gene of interest (kanamycin resistance cassette, Kan^R^) flanked with (IRs), contains a conditional origin of replication, oriR6K, which prevents replication in the recipient strain. Donor plasmid DNA and purified recombinant transposase (Mos1 or Mboumar-9) are co-transfected into bacterial cells, resulting in integration of the kanamycin cassette into the genomic DNA. (**B**) Kanamycin resistant colonies were obtained only if purified transposase was included in the transfection reaction. White colonies on dark background. (**C**) Relative efficiency of transposition observed after treatment of the reactions, prior to transfection, with proteinase K, phenol, no treatment and the control (no transposase added). Two technical repeats were performed for two biological repeats. (**D**) Agarose gel of the products of colony polymerase chain reaction (PCR) to detect the presence of the donor plasmid in the kanamycin resistant colonies. Clone 1 (Lane 4) has traces of the donor plasmid backbone detected. Positive control—a colony of *Escherichia coli* S17 λ pir carrying donor plasmid; negative control—a colony of the recipient strain *E. coli* DH10B. (**E**) Five analyzed clones are resistant to kanamycin, but sensitive to chloramphenicol—the plasmid backbone resistance. Controls as in (D). (**F**) Southern Blotting analysis of the digested genomic DNA from the kanamycin resistant clones hybridized with fluorescently labeled IR DNA. Negative control: genomic DNA of the recipient strain *E. coli* DH10B. (**G**) WebLogo alignment of 40 bp around the TA target nucleotides duplication of the 14 integration sites by Mos1 in the bacterial genome.

DNA transposition can be assayed by an *in vitro* hop assay, in which a kanamycin resistance cassette, flanked by IRs, moves from a donor to a recipient plasmid upon addition of transposase and divalent cations (39) (Figure 1). After transformation of the reaction product into *E. coli* DH10B, the donor plasmid cannot replicate since it contains the conditional origin of replication oriR6K, whereas kanamycin resistant products of transposition (genomic integrations) are selected and propagated. During optimization of this protocol for Mos1 we noticed that if transposase protein was not inactivated prior to transfection, kanamycin resistant colonies were obtained (Figure 2A and B). The number of such colonies was reduced 10-fold after incubation of the reaction products with proteinase K, and was reduced to zero by phenol extraction of the reaction mixture (Figure 2C). Even though, in some colonies a low concentration of the replication-deficient plasmid was detected by PCR (Figure 2D, clone 1), these clones were unable to grow in the presence of chloramphenicol—the resistance encoded by the donor plasmid backbone (Figure 2E). We hypothesised that the kanamycin resistant colonies could have been products of genomic integration.

We suspected that the kanamycin-resistant clones resulted from transposase:DNA complexes entering the cells during transfection, followed by integration of the kanamycin cassette into the *E. coli* genome. To investigate this, we digested the genomic DNA of the kanamycin-resistant clones and performed Southern Blot analysis by hybridization with fluorescently labeled IR DNA (28 bp). All the clones analyzed contained between one and three integrations (Figure 2F). To confirm the *in vivo* integrations, we sequenced the integration sites in genomic DNA after Mos1 and Mboumar-9 transposition (Supplementary Figure S1 and 2; Supplementary Table S1 and 2). Alignment of these sites for Mos1 transposition (Figure 2G) and for Mboumar-9 transposition (Supplementary Figure S3) showed that the integrations occurred with duplication of the TA target site, confirming that they were indeed products of *mariner* transposition (40).

**Figure 3.**
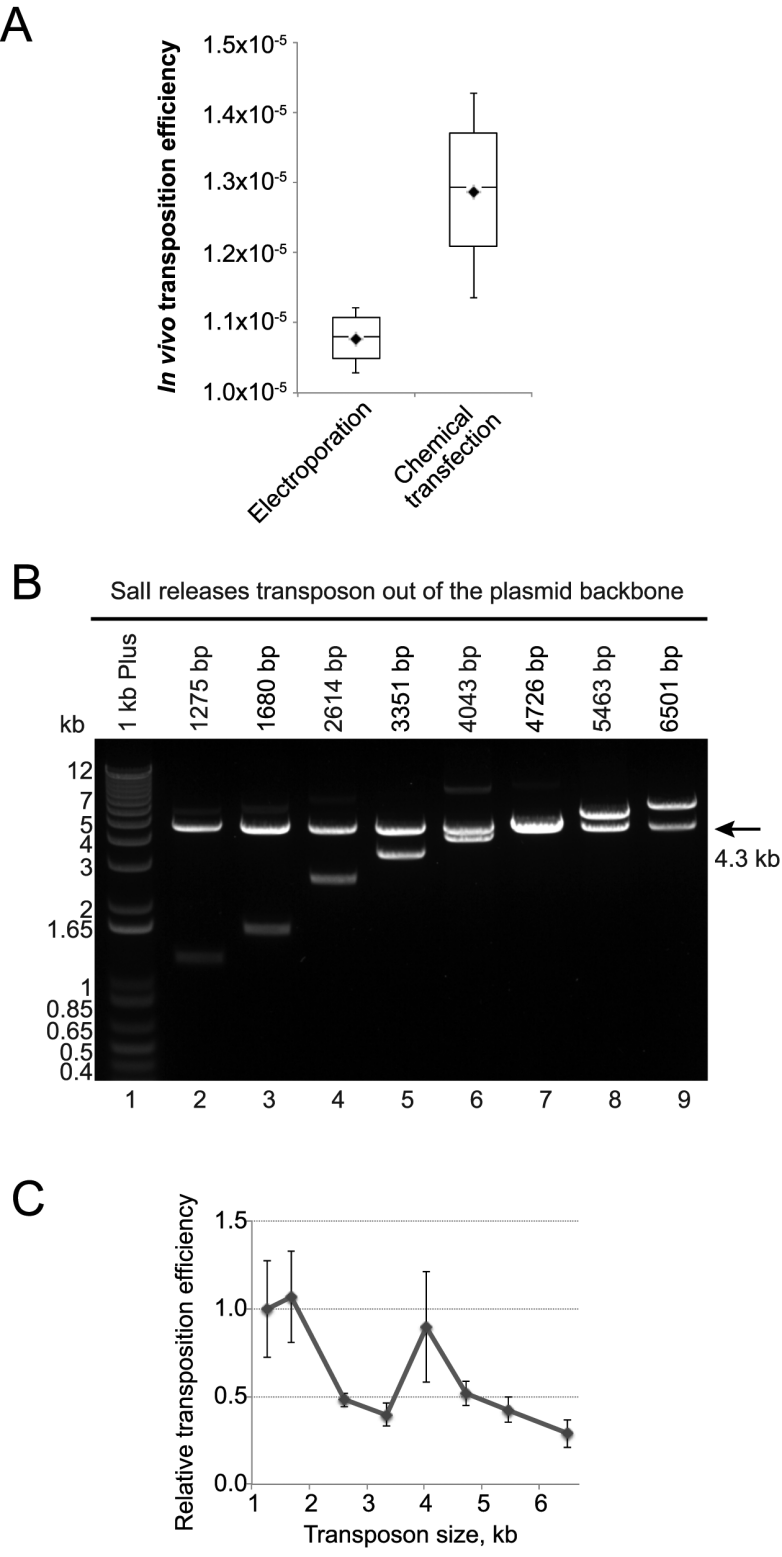
Comparison of transfection methods and transposon sizes for *in vivo* transposition. (**A**) *In vivo* transposition efficiency after electroporation or chemical transfection. *In vivo* transposition efficiency is the number of resistant colonies per microgram of the donor plasmid divided by the efficiency of transfection of a standard plasmid (CFU/μg). (**B**) Agarose gel of the restriction analysis to confirm the correct size of each plasmid donor of transposon assembled by PaperClip. (**C**) Correlation of the transposition efficiency with the transposon size after Mos1 transposition. For panels A and C two technical repeats were performed for two biological repeats.

In our experiments, both electroporation and chemical transfection of purified *mariner* transposases resulted in similar transposition efficiency in bacterial cells (Figure 3A). This observation indicates that the transfection method in not a limiting step for transposition *in vivo*.

### Size limitations for *in vivo* transposition

The efficiency of *in vitro mariner* transposition is dependent upon the separation of the IRs (19,41): previously it was observed that increasing the size of the transposon gene causes dramatic reduction in Mos1 transposition efficiency (42,43). To check the size limitation of the transposon gene for *in vivo* transposition, we created eight different transposons, ranging in size from 1.3–6.5 kb (Figure 3B and Supplementary Table S3). To confirm that the full transposon was integrated into the genome, we performed colony PCR with primers complementary to the ends of each transposon (Supplementary Figure S4). Interestingly, when comparing reactions that used equimolar amounts of the different donor plasmids, transposition efficiency decreased only 3-fold between the longest and the shortest constructs (Figure 3C). Sinzelle *et al*. observed 20-fold decrease in efficiency of transposition for Mos1 transposons of similar sizes (43). Thus, we conclude that, at least within this range, the size of the gene of interest is not a limiting factor for *in vivo* transposition.

### *In vivo* integration in mammalian genomes

**Figure 4.**
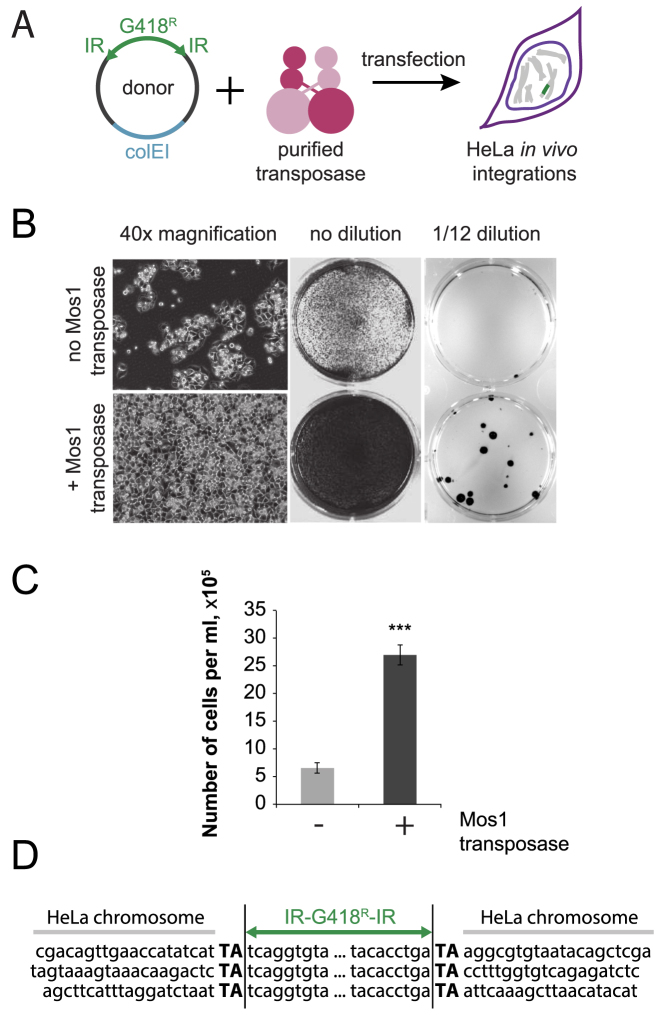
*In vivo* transposition in eukaryotic cells. (**A**) Scheme of the experimental method. The donor plasmid DNA, carrying a gene of interest (G418 resistance cassette) flanked with Mos1 IRs. Donor DNA and purified recombinant transposase are co-transfected into mammalian cells, resulting in integration of the gene of interest into genomic DNA. (**B**) HeLa cells after 11 days of selection (40× magnification), after 13 days of selection (no dilution) and after 10 days of selection (1/12 dilution). Addition of the Mos1 transposase increases the number of G418-resistant cells. Three biological repeats. (**C**) Quantification of the number of G418 resistant cells. Data are represented as average of six repeats (two technical repeats for three biological repeats) ± standard deviation. *** *P-*value < 0.001. (**D**) Localization of the integrations in mammalian genome. TA dinucleotides were duplicated upon transposon DNA integration.

Next we asked if *in vivo* transposition could be observed in mammalian cell lines. The G418 resistance gene was flanked with IRs of Mos1 to create a donor plasmid (Figure 4A). We found that after transfection of HeLa or HEK293-H cells with donor plasmid together with purified transposase, we obtained an increased number of G418 resistant colonies, compared to the no-transposase control (Figure 4B, Supplementary Figure S5). In the HeLa cell line after addition of purified transposase, the number of G418 resistant cells increased on average by a factor of 5 (Figure 4B, no dilution, 4C). The number of G418 resistant colonies increased from 2 ± 0.7 (no transposase added) to 11 ± 4 (transposase added) (Figure 4B, 1/12 dilution). Three integration sites in the HeLa genome were sequenced directly: duplication of the TA target dinucleotides confirmed that the insertions were products of a *mariner* transposition reaction (Figure 4D). In the HEK293-H cell line the number of G418 resistant colonies increased two times from 36 ± 7 (no transposase added) to 67 ± 10 (transposase added) (Supplementary Figure S5, 1/6 dilution). The difference between HeLa and HEK293-H cell lines response to *in vivo* transposition might be due to several factors including but not limited to higher transfection or higher recombination efficiencies of the HEK293-H cell line.

**Figure 5.**
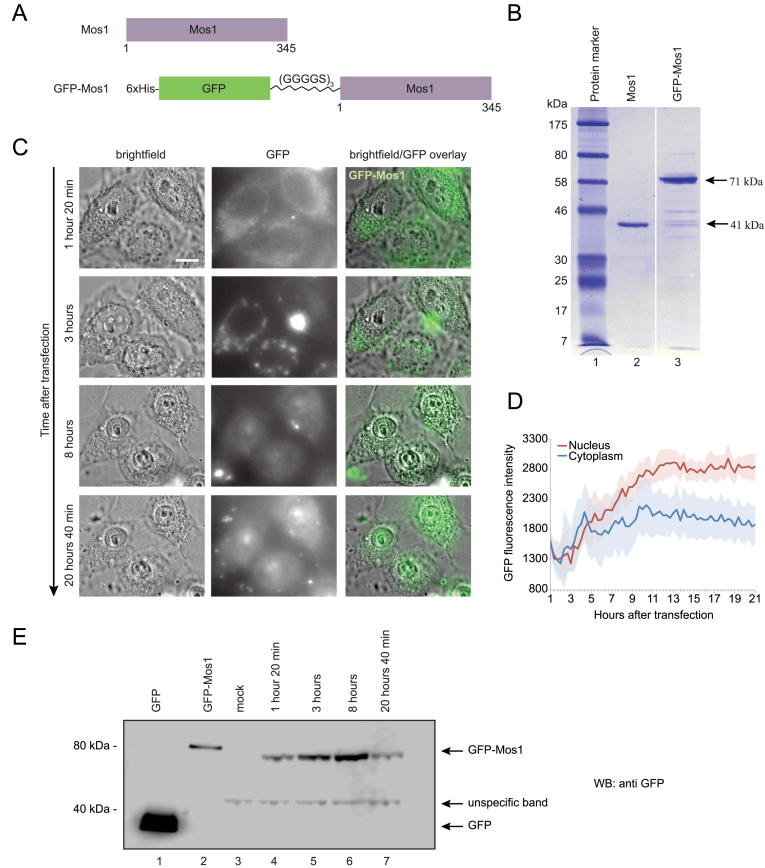
Construction and imaging of GFP-Mos1 fusion transposase. (**A**) Mos1 and GFP-Mos1 transposase fusion protein constructs in pET30a expression vector. (**B**) Coomassie stained 12% SDS-PAGE in Tris-Glycine-SDS buffer of purified Mos1 and GFP-Mos1 transposases. (**C**) Live cell imaging with 60× magnification after transfection of HeLa cells with purified GFP-Mos1 transposase in complex with donor DNA. Scale bar is 10 μm. Images are representatives of 10 image points on a dish. (**D**) Quantification of the GFP signal in the nucleus and cytoplasm over time after transfection; 10 cells were analyzed. Shadowed error bars represent the standard deviation. (**E**) Western blotting analysis of HeLa cells lysates after transfection with GFP-Mos1 and donor plasmid. Proteins were separated on 4–12% NuPAGE SDS-PAGE in MOPS buffer (buffer composition, acrylamide percentage and protein concentration affect protein migration). Lane 1–1 ng of purified GFP, lane 2–10 ng of purified GFP-Mos1, lane 3-mock, no transposase or DNA was added to the cells. In lanes 3–7, 250 μg of total HeLa cell extracts were loaded. Lanes 4–7 contain full-length GFP-Mos1 transposase with no evidence of protein degradation 20 h post transfection. The antibody against GFP recognizes an unspecific protein, which is present in all time points and the mock control. Lane 7 shows lower signal for GFP-Mos1 protein since the cells have undergone division, which dilutes GFP-Mos1 concentration versus total protein concentration.

In order to determine whether the transposase can indeed penetrate the mammalian cell membrane, we expressed and purified an N-terminal GFP-Mos1 transposase fusion with a flexible linker (GGGGS)_3_ (Figure 5A and B). Introduction of the GFP cassette did not reduce the efficiency of prokaryotic transposition *in vivo* when compared to unfused Mos1 transposase (Supplementary Figure S6).

During imaging of live cells, the transposase was visible in the cell cytoplasm 80 min after transfection (Figure 5C). After 3 h, the transposase protein formed aggregates in the cytoplasm, and after 8 h, it penetrated the nuclear envelope. By 20 h post-transfection, the highest transposase concentration was in the nucleus, where *in vivo* transposition can occur (Figure 5D). To confirm that the GFP signal in the nucleus is not due to diffusion of the GFP on its own, in the event of GFP-Mos1 degradation inside the cells, we performed a western blotting analysis of the HeLa cell extracts, prepared at the same time points (Figure 5E). Our results indicate that there is no detectable GFP-Mos1 degradation 20 h post-transfection. This is a direct confirmation that recombinant Mos1 transposase, expressed and purified from *E. coli*, can indeed penetrate mammalian cells during transfection and concentrate in the nucleus retaining its integrity.

## DISCUSSION

Taken together, our results show that two transposases of the *mariner* family are able to facilitate integration of target DNA into the genome of living prokaryotic or eukaryotic cells *in vivo*, without expression of the transposases inside the host cells. Transposase together with transposon DNA were successfully introduced into bacterial or mammalian cells by electroporation or transfection. It has been shown that co-transfection of restriction enzymes increases the recombination rate in eukaryotic cells (44,45), but to the best of our knowledge, chemical transfection of transposases has not been reported to date. Genomic integration has been observed previously after the integration products of Tn5 (32) or Mu (46) transposition *in vitro* were introduced into cells by electroporation. Electroporation of proteins into cells is a well known procedure (47); however, we are not aware of any published protocols which introduce active transposons into cells via methods other than electroporation or injection. The ability to function in cells following chemical transformation/transfection, at similar efficiencies to those seen with electroporation, may be unique to *mariner* transposases, or it may be that other systems such as EZ-Tn5^™^ are also effective following chemical transformation procedures. Electroporation is difficult to automate; thus protocols, which do not require electroporation offer the potential for automated high-throughput generation of insertion libraries. *Mariner* transposases can integrate transposons in any TA dinucleotide present in the genome (36,48), providing an advantage for full genome coverage.

As with other protein-based transposase systems, this method allows screening of prokaryotic and eukaryotic host organisms without the need for specific promoters, vectors or delivery tools (49). This is a great advantage for biotechnology, which is seeking alternative chassis for production of valuable compounds. Our method provides an IP-free tool to deliver and integrate genes and pathways of interest into prokaryotic and eukaryotic genomes. Therefore the time for screening for the ideal organism, strain and position of stable integration can be reduced dramatically. Nevertheless, it should be noted that different organisms and cell lines might require optimisation of protein concentration, transfection reagent and selection marker.

Moreover, as our method does not require DNA encoding the transposase, it overcomes the problem of unstable integration in the host cells, due to prolonged transposase expression. The absence of the transposase gene precludes its recombination with the host DNA, which could result in constant expression of transposase leading to undesired genomic rearrangements. Since the transposase concentration can be finely tuned by titration, overproduction inhibition should not be a limitation for *mariner* transposition *in vivo*.

In summary, Mos1 and Mboumar-9 offer a functional alternative to other widely used commercial systems such as EZ-Tn5^™^, with the added advantage that electroporation is not required, opening the possibility for automated high-throughput integration systems. The one-step method described here is an open source tool, which has the potential to be universally applied to facilitate research in basic microbiology, molecular biology and biotechnology, as well as providing a one-step gene delivery and integration tool for the generation of libraries, sequencing and gene therapy.

## Supplementary Material

Supplementary Data
