# Supplementary material for: Use of mariner transposases for one-step delivery and integration of DNA in prokaryotes and eukaryotes by transfection
Source: Nucleic Acids Res. 2017 Feb 16;45(10):e89. doi: 10.1093/nar/gkx113 (PMC5449632; doi:10.1093/nar/gkx113)
Supplement: Supplementary Data [file gkx113_supp.pdf]

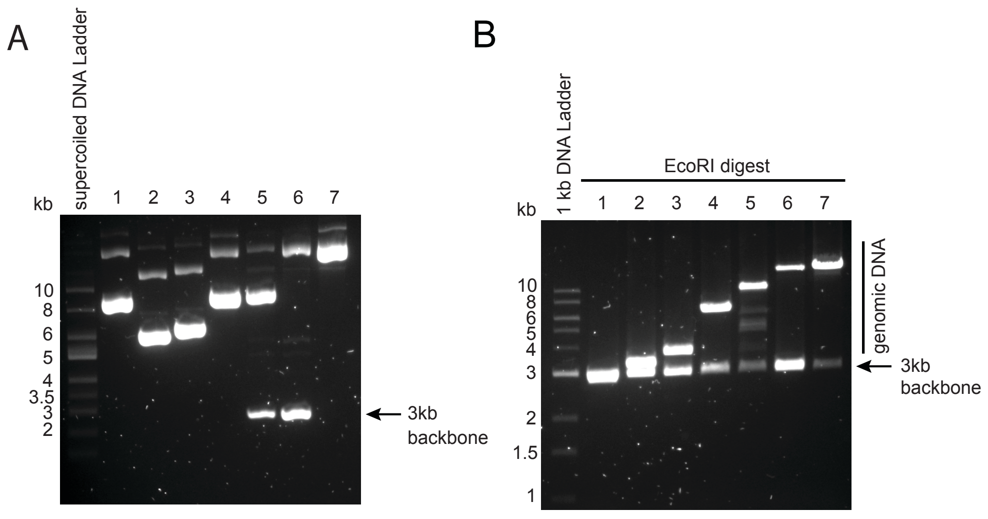


**Supplementary Figure 1.** Localization of the genomic transposon integrations in bacterial cells by cloning of genomic DNA. (**A**) Agarose gel analysis of plasmid DNA containing the pBSKS(+) backbone with cloned in by EcoRI sites fragments of genomic DNA isolated from kanamycin resistant clones. Clones 1-4 are obtained after Mboumar-9 *in vivo* transposition and clones 5-7 are obtained by Mos1 mediated *in vivo* transposition. (**B**) Restriction digestion analysis of these plasmids with EcoRI, which releases the genomic DNA insert and free pBSKS(+) backbone. Plasmid DNA was sequenced by the Sanger method.

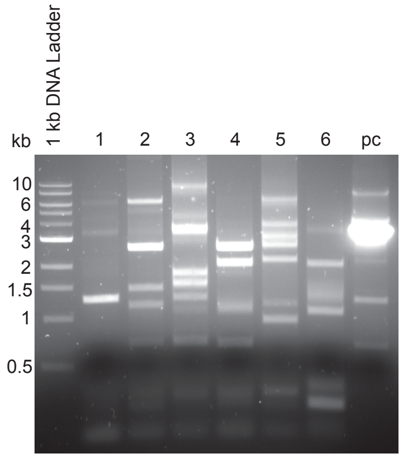


**Supplementary Figure 2.** Localization of the genomic transposon integrations into bacterial cells by inverted PCR. Inverted PCR was performed on self-ligated genomic DNA after *in vivo* transposition by Mboumar-9 transposase. Positive control (pc) plasmid donor of transposon, expected product 4.3 kb. Selected bands were purified from the agarose gel and subjected to Sanger sequencing.

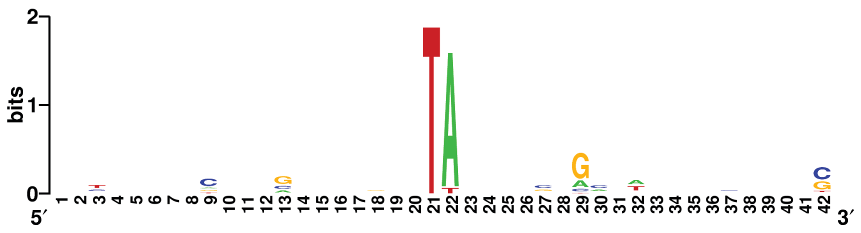


**Supplementary Figure 3.** Alignment of 20 integration sites by Mboumar-9 reveals no preferential consensus around duplicated TA bases.

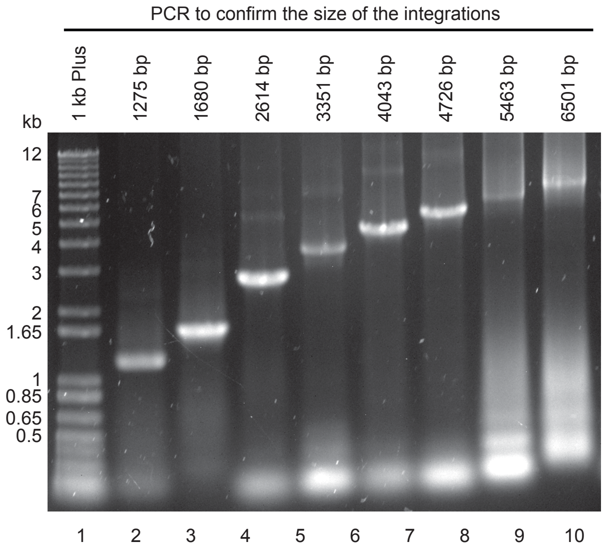


**Supplementary Figure 4.** PCR on isolated genomic DNA from kanamycin resistant colonies to confirm the size of the integrated constructs. The full-length transposons were integrated.

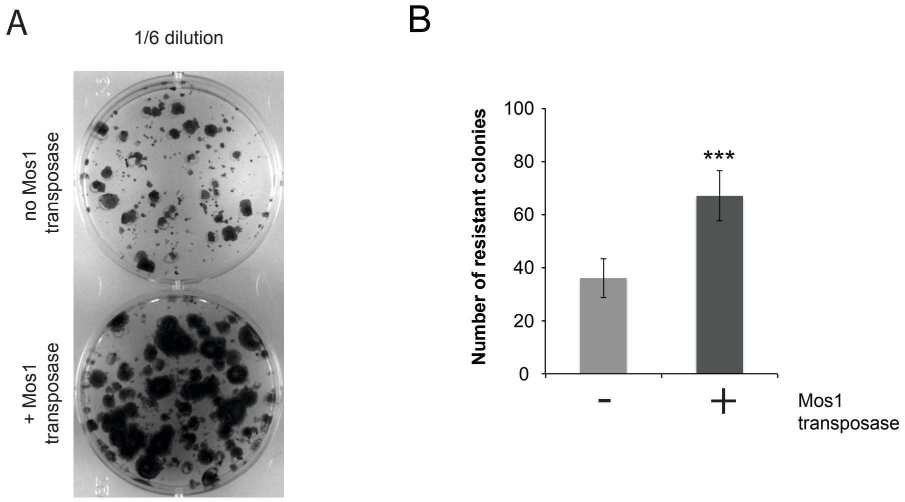


**Supplementary Figure 5.** Mos1 mediated *in vivo* transposition in HEK293-H cells. (**A**) HEK293-H cells after 13 days of selection in G418-containing medium. Addition of Mos1 transposase results in increased number of G418 resistant colonies. Two biological and two technical replicas were performed for the control reactions without Mos1 transposase. Three biological and two technical replicas were performed for the reactions where Mos1 transposase was aded. (**B**) Quantification of the number of G418 resistant colonies of HEK293-H cells. Error bars are standart deviation, *** p-value < 0.001.

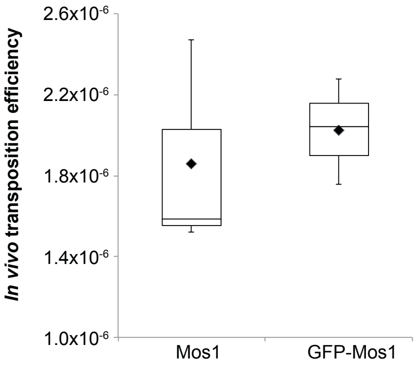


**Supplementary Figure 6.** *In vivo* transposition in *E.coli* DH10B to test the activity of GFP-Mos1 fusion protein, protein:DNA ratio is 20:1. The 1.3 kb kanamycin cassette transposon was used in this experiment. Efficiency of transposition is number of kanamycin resistant colonies devided by the efficiency of transformation ($4.41x10^{-7}$ CFU/ug). Two technical repeats were performed for each of three biological repeats in this experiment.

| | *E.coli* genomic DNA | | IR-Kan$^R$-IR | | | | *E.coli* genomic DNA |
|---|---|---|---|---|---|---|---|
| 1 | tgtggacaatattgtcgaac | TA | tcaggtgta | … | tacacctga | TA | gcgcgtaagcctggtctgag |
| 2 | ctggatgacacgcagctgcg | TA | tcaggtgta | … | tacacctga | TA | atctggaaacgcgtctgagc |
| 3 | ccccccgcgacaatcaacgg | TA | tcaggtgta | … | tacacctga | TA | cggcaaaagagattccgctc |
| 4 | tcataggtctgctcaagccg | TA | tcaggtgta | … | tacacctga | TA | attgatatttgtcaatttta |
| 5 | ggtatggtcttcagcgcgct | TA | tcaggtgta | … | tacacctga | TA | aaggtaagccaatcagttcg |
| 6 | gatcctcagcgaggatgacg | TA | tcaggtgta | … | tacacctga | TA | acgataacacataaagtaaa |
| 7 | agcttccactttaggcagca | TA | tcaggtgta | … | tacacctga | TA | gaacctttcgcaaaatgacc |
| 8 | tgccagcgactgtgaagtat | TA | tcaggtgta | … | tacacctga | TA | atgtcaggtattgccgacgg |
| 9 | tgataaattgcgtcagaaag | TA | tcaggtgta | … | tacacctga | TA | cccggtttacgtaacgttgc |
| 10 | tctctgcgccaatgccaaac | AA | tcaggtgta | … | tacacctga | AA | tttgctctcaacggcgtcga |
| 11 | ctggcaacttcttagtgccg | TA | tcaggtgta | … | tacacctga | TA | atgatgtttctggccagcac |
| 12 | caacaggtttcgtgacggct | TA | tcaggtgta | … | tacacctga | TA | tggaacccgccgacgctcaa |
| 13 | acccgcgatgttaagcgcga | TA | tcaggtgta | … | tacacctga | TA | cgctgcccggcattggcggt |
| 14 | caacaggtttcgtgacggct | TA | tcaggtgta | … | tacacctga | TA | ctggaacccgccgacgctca |

**Supplementary Table 1.** Mos1 integration sites in *E.coli*. Sequences of 14 genomic integrations mediated by Mos1.

| | *E.coli* genomic DNA | | IR-Kan$^R$-IR | | | | *E.coli* genomic DNA |
|---|---|---|---|---|---|---|---|
| 1 | aactggaagcagacgtcgcg | TA | ccaggagtg | … | cactcctgg | TA | cgtccagggcacgttcttgc |
| 2 | ggtatgcgcgccagagtgat | TA | ccaggtgtg | … | cacacctgg | TA | atgcaggattttcgcgagga |
| 3 | cgttttcccgctgtgatagc | TA | ccaggtgtg | … | cactcctgg | TA | cccttaaagactgactcttt |
| 4 | tttgataccaccgaacggga | TA | ccaggtgtg | … | cactcctgg | TA | agagcacgtttcagcggagc |
| 5 | tatgtctgtgaccgcggggg | TA | ccaggtgtg | … | cacacctgg | TA | tatggtgctgctggccattc |
| 6 | gttaaagcattaggcgggga | TA | ccaggtgtg | … | cacacctgg | TA | tgcaggccgagctgattgac |
| 7 | gatttcagcaaacagctgca | TA | ccaggtgtg | … | cacacctgg | TA | ccgtccatttcatccatccg |
| 8 | ttattggtgaaggtgatgca | TA | ccaggtgtg | … | cacacctgg | TA | tattccgcctcatacaggtc |
| 9 | caccgcctgcatggtggtaa | TA | ccaggtgtg | … | cacacctgg | TA | gaggcagagaagttttcgct |
| 10 | ccgcccaaagtagtcaccaa | TA | ccaggtgtg | … | cacacctgg | TA | aaacccaccagtaagtgagc |
| 11 | acttgcgactttggctgctt | TT | ccaggtgtg | … | cacacctgg | TT | tgtatggtgaaagatgtgcc |
| 12 | gtgcccgatcacctgaaaat | TA | ccaggtgtg | … | cacacctgg | TA | ccttccgcgtggtggatgac |
| 13 | atagcaccaggcgatggtta | TA | ccaggtgtg | … | cacacctgg | TA | ctgtgtggctcccggtcccg |
| 14 | attcagctcgccatcggcga | TA | ccaggtgtg | … | cacacctgg | TA | tgctgatcaatctgcgccag |
| 15 | cgccgcgcccctgaactttg | TA | ccaggtgtg | … | cacacctgg | TA | gccaccgaaaatattcactg |
| 16 | cggaccgccgcgcgcccatt | TA | ccaggtgtg | … | cacacctgg | TA | ccgtggaagtagccatcttc |
| 17 | agtgatcgcctttttaccgg | TA | ccaggtgtg | … | cacacctgg | TA | aggactgcaagttccaggtg |
| 18 | ttctgatatcaggcgatgac | TA | ccaggtgtg | … | cacacctgg | TA | tgtggtgcatcgccaaccgc |
| 19 | cgcaccaacaaacttagcaa | TA | ccaggtgtg | … | cacacctgg | TA | gcggttgcccggtgcgtttg |
| 20 | cgctgtcacagaaccgagac | TA | ccaggtgtg | … | cacacctgg | TA | tcaaatgaaggatttaaacg |

**Supplementary Table 2.** Mboumar-9 integrations sites in *E.coli*. Sequences of 20 genomic integrations mediated by Mboumar-9.

| Construct | Size, bp | Source | Comments |
|---|---|---|---|
| Kan | 1275 | (36) | |
| Kan-pLacLacZ | 1680 | (37) | |
| pLacLacZ-Kan-Amp | 2614 | | |
| pLacLacZ-GFP-Kan-Amp | 3351 | | |
| pLacLacZ-RFP-GFP-Kan-Amp | 4043 | | |
| pLacLacZ-RFP-GFP-Kan-amilCP-Amp | 4726 | assembled in this study | |
| pLacLacZ-RFP-GFP-Kan-amilCP-YFP-Amp | 5463 | | |
| pLacLacZ-RFP-GFP-Kan-Mos1-amilCP-YFP-Amp | 6501 | | deletion in YFP |

**Supplementary Table 3.** Parts included into each of eight transposons constructs.

| | | | |
|---|---|---|---|
| 1 | Mos1 IR, 28-mer | aaacgacatttcatacttgtacacctga | 5' IRDye700 modification, for Southern Blotting |
| 2 | KanFor | gtttcccgttgaatatggctc | sequencing of flanking DNA after transposition, inverted PCR |
| 3 | KanRev | actttctggctggatgatgg | sequencing of flanking DNA after transposition, inverted PCR |
| 4 | YFP_UF | gccaggaaacagctatggtgagcaagggcgaggagctgttcac | PaperClip assembly |
| 5 | YFP_UR | aacagctcctcgcccttgctcaccatagctgtttcct | PaperClip assembly |
| 6 | YFP_DF | ccgggatcactctcggcatggacgagctgtacaagtaataa | PaperClip assembly, sequencing |
| 7 | YFP_DR | ggcttattacttgtacagctcgtccatgccgagagtgatcc | PaperClip assembly, sequencing |
| 8 | amilCP_UF | gccaggaaacagctatgagtgtgatcgctaaacaaatgacctac | PaperClip assembly |
| 9 | amiCP_UR | ggtcatttgtttagcgatcacactcatagctgtttcct | PaperClip assembly |
| 10 | amilCP_DF | tgaaatttccattgcacgcaaacctgtggtcgcctaataa | PaperClip assembly |
| 11 | amilCP_DR | ggcttattaggcgaccacaggtttgcgtgcaatggaaatt | PaperClip assembly |
| 12 | Mos1_UF | gccagcagctttgtgccgaacaaagaacagacccgc | PaperClip assembly |
| 13 | Mos1_UR | ggtctgttctttgttcggcacaaagctgct | PaperClip assembly |
| 14 | Mos1_DF | ggaaaaatgcgtggcgagcgatggcaagtactttgaataa | PaperClip assembly, sequencing |
| 15 | Mos1_DR | ggcttattcaaagtacttgccatcgctcgccacgcatttt | PaperClip assembly |
| 16 | KanF_SalI | atttatgtcgaccgctgaggtctgcctcg | amplification of the cassettes assembled in pSBC3, introducing SalI site |
| 17 | LacR_SalI | ttaaatgtcgacggatccaggctcatccagcc | amplification of the cassettes assembled in pSBC3, introducing SalI site |
| 18 | LacF_SalI | ttaaatgtcgactctagattcggagtgagc | amplification of the cassettes assembled in pSBC3, introducing SalI site |
| 19 | AmpR_SalI | taagatgtcgacttcaaatatgtatccgctcatg | amplification of the cassettes assembled in pSBC3, introducing SalI site |
| 20 | SalI_Neo_For | tccatagtcgacagtcctgaggcggaaagaacc | amplification of NeoR cassette from pEGFP-N1, introducing SalI site |
| 21 | SalI_Neo_Rev | tccatagtcgacatgagtaacctgaggctatggc | amplification of NeoR cassette from pEGFP-N1, introducing SalI site |
| 22 | pET30a_UF | gcctaataactcgagcaccaccaccaccaccactgagatccgg | PaperClip assembly |
| 23 | pET30a_UR | gatctcagtggtggtggtggtggtgctcgagttatta | PaperClip assembly |
| 24 | pET30a_DF | tcatcattcttctggtctggtgccacgcggttctggtatg | PaperClip assembly |
| 25 | pET30a_DR | ggccataccagaaccgcgtggcaccagaccagaagaatga | PaperClip assembly |
| 26 | GFPfus_UF | gcccgtaaaggagaagaacttttcactggagttgtcccaattc | PaperClip assembly |
| 27 | GFPfus_UR | ttgggacaactccagtgaaaagttcttctcctttacg | PaperClip assembly |
| 28 | GFPfus_DF | agctgctgggattacacatggcatggatgaactatacaaa | PaperClip assembly, sequencing |
| 29 | GFPfus_DR | ggctttgtatagttcatccatgccatgtgtaatcccagca | PaperClip assembly |
| 30 | Linker2_UF | gccggtggcggcggtagcggtggtggc | (GGGGS)3 flexible linker, PaperClip assembly |
| 31 | Linker2_UR | gaaccgccaccaccgctaccgccgccacc | (GGGGS)3 flexible linker, PaperClip assembly |
| 32 | Linker2_DF | ggttctggcggtggcggcagc | (GGGGS)3 flexible linker, PaperClip assembly |
| 33 | Linker2_DR | ggcgctgccgccaccgcca | (GGGGS)3 flexible linker, PaperClip assembly |
| 34 | VF2 | tgccacctgacgtctaagaa | sequencing |
| 35 | VR | attaccgcctttgagtgagc | sequencing |
| 36 | RFPmidF | ctacctgaaactgtccttcc | sequencing |
| 37 | pEP_noSalI_F | ccctcgaggtagacggtatcgataagc | deletion of SalI site from pEP185.2 vector |
| 38 | pEP_noSalI_R | gcttatcgataccgtctacctcgaggg | deletion of SalI site from pEP185.2 vector |
| 39 | pBS_noSalI_F | gcttatcgataccgacgacctcgaggggg | deletion of SalI site from pBSKS(+) vector |
| 40 | pBS_noSalI_R | ccccctcgaggtcgtcggtatcgataagc | deletion of SalI site from pBSKS(+) vector |

**Supplementary Table 4.** DNA oligonucleotides used in this study.
